# Vehicle driving area detection and sensor data preprocessing based on deep learning

**DOI:** 10.1371/journal.pone.0337722

**Published:** 2025-12-16

**Authors:** Jun Zhou, Nuo Xu, Xuexuan Wu

**Affiliations:** Faculty of Transportation Engineering, Huaiyin Institute of Technology, Huai’an, China; Sunway University, MALAYSIA

## Abstract

With the rapid development of intelligent vehicles, it has become particularly important to effectively detect the environment of the vehicle’s driving area. A vehicle driving road recognition algorithm on the basis of an improved bilateral segmentation network is built to address the poor real-time performance and low accuracy in current intelligent vehicle driving area detection methods. Combined with the algorithm, a vehicle driving area detection model based on bilateral segmentation network and data dimensionality reduction is designed. The performance comparison analysis between the recognition algorithm and other algorithms showed that the average frame processed per second and the average recognition time were 68.78FPS and 4.45ms, outperforming the comparison algorithms. The average precision and accuracy were 98.97% and 97.66%, both higher than the comparison models. Finally, the application effect analysis showed that it had good detection performance. The proposed recognition algorithm and detection model have effectiveness and practical value, which can help improve the real-time and accuracy of intelligent vehicle driving area detection, and provide theoretical basis for related research.

## 1. Introduction

Driven by science and technology, utilizing deep learning technology to achieve automotive intelligence has become an inevitable trend in automotive industry [1]. The vehicle is equipped with sensors to collect environmental data around the driving area. Moreover, using deep learning techniques to analyze these data can achieve region detection, which is an important guarantee for safe driving [2]. Road surface detection is a key part of area detection during vehicle operation. Effective road surface detection is conducive to providing a basis for the trajectory planning of autonomous vehicle, so as to achieve safe driving [3]. However, the current road surface detection methods for vehicle driving areas suffer from poor real-time performance. Many experts have explored, but the results are still lacking. Y. Ai et al. built a road boundary detection framework on the basis of meta-learning and random forest to solve the low accuracy in road boundary detection in intelligent vehicle driving areas. Although it improved the accuracy, the real-time performance was poor [4]. Regarding the real-time detection of intelligent vehicle environment, Z. Wang et al. used a literature review method to review the sensors and algorithms for intelligent vehicle detection over the years. Although they have certain effects, they did not propose practical methods and have no practical value [5]. S. Yang et al. put forward a constructive strategy to improve the intelligent tire sensing system to solve the poor real-time performance of current road condition recognition methods for autonomous vehicle. However, it only existed in the theoretical stage and was not practical [6]. Some studies have some achievements. T. Luo et al. built a unified multi-task network for road scenarios to solve the high energy consumption by intelligent vehicles in road environments. The network was effective [7]. Autonomous vehicle cannot accurately detect road information anomalies. Therefore, C. Liu proposed a responsive road anomaly detection and evaluation method based on autonomous vehicle ‘vibration data acquisition. The method had good detection effect [8].

Deep-learning technology is rapidly on the rise, and applying it to road surface detection in intelligent vehicle driving areas is significant for the safe driving. Bilateral Segmentation Network (BiSeNet) is a deep learning network used for semantic segmentation tasks, which has advantages such as high efficiency and accuracy [9]. You Only Look Once version 5 (YOLOv5) is a deep learning based object detection algorithm that has fast detection speed and high accuracy. It is largely applied in fields such as object detection and road defect detection [10]. Many experts have conducted relevant research. Y. Sun et al. built an intelligent vehicle adaptive detection based on BiSeNet to solve the intelligent vehicles being unable to accurately detect the number of lanes. Compared with similar methods, this method was more robust [11]. In response to the poor real-time performance in intelligent vehicle road segmentation, Y. Wangu et al. built a semantic segmentation model relying on BiSeNet, which was experimentally verified to be effective [12]. Z. Zhang et al. built a detection method relying on YOLOv5 to solve the low accuracy in object detection in autonomous driving areas. Performance evaluation experiments were conducted on this method. It outperformed previous detection methods [13]. Mahaur et al. adopted an improved YOLOv5 to address the intelligent vehicles being unable to accurately recognize traffic signs in low light and adverse weather conditions. The model was compared and analyzed with traditional models. The results showed that it had higher detection accuracy [14]. In response to the low accuracy in target detection of intelligent vehicles in foggy weather, G. Li et al. designed an improved YOLOv5, and conducted experiments on different public driving datasets. The method proved to be good at detecting [15].

The above research results indicate that the current methods applied to intelligent vehicle driving area detection have poor real-time performance and low accuracy. Therefore, the study emphasize the irregular road areas encountered during vehicle driving, and introduces improved ResNet18 network, Efficient Channel Attention (ECA) mechanism, Global Convolution Network (GCN), and Boundary Refinement (BR) to improve it. A vehicle driving road recognition algorithm based on improved BiSeNet is constructed. Secondly, an improved YOLOv5 algorithm is introduced to construct a road pothole detection algorithm based on the improved YOLOv5. The Principal Component Analysis (PCA) is taken to lower the data dimensionality. Finally, combining the three, a vehicle driving area detection method based on BiSeNet and data dimensionality reduction is constructed, aiming to optimize the real-time performance of vehicle driving area detection. The innovation of this study lies in combining improved BiSeNet, improved YOLOv5 algorithm, and PCA-based sensor data dimensionality reduction method to provide a theoretical basis for research on intelligent vehicle driving.

## 2. Methods and materials

### 2.1. Design of vehicle driving road recognition algorithm based on improved bisenet

**Fig 1:**
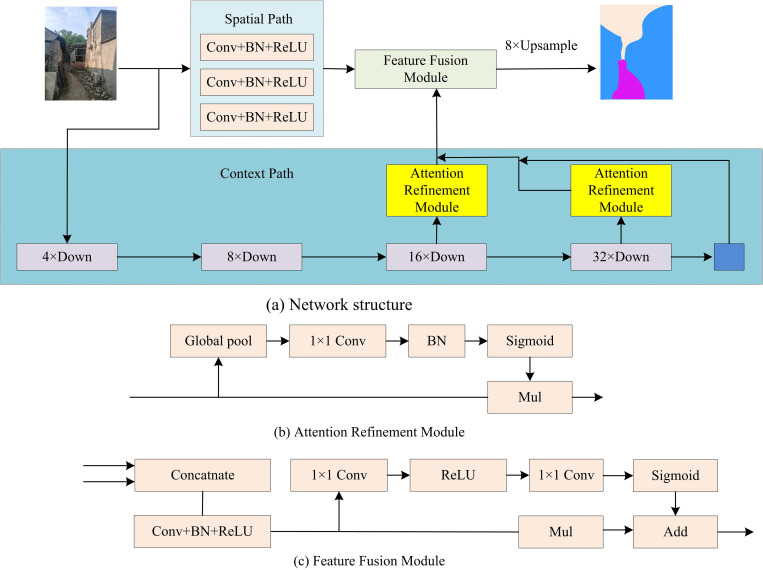
BiSeNet structure (Source from: Photo by author).

In recent years, autonomous driving and intelligent car assistance systems have developed rapidly. However, when facing informal roads, existing road segmentation methods are difficult to achieve high accuracy due to unclear road boundaries and irregular shapes, resulting in inaccurate road recognition. To solve this problem, the BiSeNet is introduced and improved ResNet18 network, ECA, GCN, and BR are introduced to construct a vehicle driving road recognition algorithm based on the improved BiSeNet. BiSeNet is used for real-time semantic segmentation, with high efficiency and accuracy. Its application is extensive, spanning fields like autonomous driving and augmented reality [16]. Fig 1 displays the BiSeNet structure [17].

**Fig 2:**
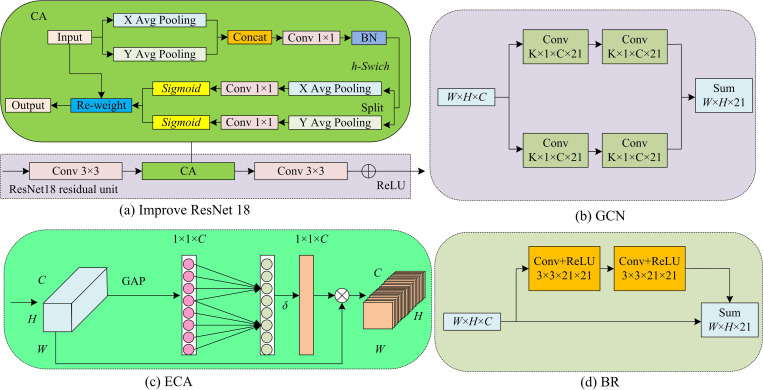
Basic network structures of ResNet18 network, ECA, GCN and BR.

From Fig 1, the BiSeNet mainly has three parts: spatial path, contextual path, and feature fusion module. The main purpose of spatial paths is to retain spatial information and produce high-resolution feature maps. It has three convolutional layers (Conv) + Batch Normalization (BN) + ReLU activation function modules. The contextual path is to obtain sufficient contextual information, that is, an objective receptive field. It integrates features from contextual paths through attention refinement modules to extract segmentation accuracy. The feature fusion module adopts channel attention mechanism, which adaptively modify the importance of each channel in the feature map by learning the correlation between channels, thus achieving more effective feature fusion. The BiSeNet spatial path has a total of 3 layers. The convolution kernel size of the first layer is 7 7, and the convolution kernel size of the last two layers is the same, 3 3. Each layer must go through a 2-step convolution Batch normalization and a Relu nonlinear activation operation are input through this path. The output image size is 1/8 of the original image. The context path provides the network with sufficient receptive fields and semantic information. It can retain high-quality image spatial information and improve the segmentation accuracy of road areas. However, the BiSeNet has drawbacks such as loss of spatial details, limited receptive field, and poor performance in multi-branch information fusion. To address this defect and improve the recognition accuracy, an improved ResNet18 network, ECA, Coordinate Attention (CA), GCN, and BR are introduced to improve it. The basic network structures of ResNet18, CA, ECA, GCN, and BR are shown in Fig 2.

In Fig 2(a), the improved ReNet 18 network is achieved by introducing CA into the residual module of the ReNet 18 network. CA is a classic deep convolutional neural network pattern that has advantages such as simple structure and improved network performance. It is applied in image classification and semantic segmentation [18]. The ReNet 18 network consists of multiple stages, each stage containing several residual blocks. Each residual block has two convolution operations and a ReLU activation function [19]. Introducing CA into the residual module of ReNet 18 network can enhance its feature expression ability and classification accuracy. The CA obtains feature information from both the width W and height H directions of the input feature map through AP, and outputs a feature map of size C×H×1. The feature map $A_{c}^{h}(h)$ obtained by AP in the W direction can be represented by formula (1).


$$A_{c}^{h}(h)=(1/W)\sum_{0\leq 1\leq W}x_{c}(h,i)$$ (1)

In equation (1), $x_{c}(h,i)$ signifies the pixel value of the input feature map at channel c, height h, and width i. The feature map $A_{c}^{h}(w)$ obtained by AP in the H direction can be represented by formula (2).


$$A_{c}^{h}(w)=1/H\sum_{0\leq 1\leq H}x_{c}(j,w)$$ (2)

In equation (2), $x_{c}(j,w)$ signifies the pixel value of the input feature map at channel c, height h, and width w. After AP, the features are concatenated through an 1×1 convolutional layer for feature fusion. After the fusion features are calculated using the h−Swish activation function and Sigmoid activation function, they are finally re-weighted to obtain the output result of CA. The output $y_{c}(i,j)$ of CA can be represented by formula (3).


$$y_{c}(i,j)=x_{c}\times g_{c}^{h}(i)\times g_{j}^{w}(j)$$ (3)

In equation (3), $g_{j}^{w}(j)$ represents the channel attention weight obtained by AP and processing in the H direction, the value at channel c and position j. $g_{c}^{h}(i)$ signifies the channel attention weight obtained by AP and processing in the W direction, the value at channel c and position i. Fig 2(b) displays the GCN, which enhances the boundaries through two residual structures with K × 1 × C × 21. GCN is specifically designed for processing graph structured data, which can handle irregular graph structured data and large-scale graph data, with extensive application scenes, including computer vision and graph data modelling. The GCN can reduce model parameters and increase the receptive field, which helps improve semantic segmentation capabilities [20]. In Fig 2(b), C signifies the quantity of channels and K signifies the convolution kernel size. Fig 2(c) shows the specific structure of ECA, which compresses the spatial features through global Average Pooling (AP). After compression, the convolutional layer with a size of 1 × 1 is used to convolve feature maps of different sizes. The convolution kernel of ECA convolutional layer has adaptive function. When the input feature map is large, it will automatically select the convolutional layer with larger convolution kernel for convolution operation. If the input feature map is small, a convolutional layer with a smaller kernel is selected for convolution operation [21]. The adaptive function of the convolution kernel is presented in formula (4).


$$Q=\varphi (C)={\left|\left((log_{2}(C))/\alpha )+(\beta /\alpha \right)\right|}_{odd}$$ (4)

**Fig 3:**
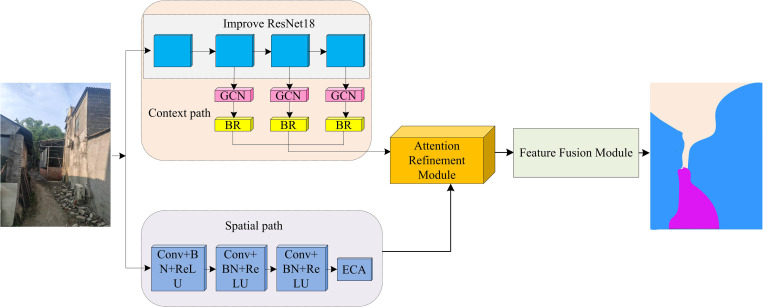
Shows the road recognition algorithm based on the improved BiSeNet (Source from: Photo by author).

In equation (4), Q signifies the calculated convolution kernel size. φ(C) signifies an adaptive function for the convolution kernel size. α signifies a hyper-parameter for adjusting the convolution kernel size, with a value of 2. β adjusts the offset of the convolution kernel size with a value of 1. Fig 2 (d) displays the BR module. BR is a module designed to improve the object boundary location accuracy. It has high flexibility and efficiency, and is widely used in auto drive system. BR utilizes large convolution kernels to capture global contextual information in images, thereby enhancing boundary information and improving the accuracy and robustness of boundary detection [22]. Therefore, a road recognition algorithm on the basis of improved BiSeNet is built, as displayed in Fig 3.

From Fig 3, the algorithm replaces the backbone network of the context path in the BiSeNet with an improved ResNet 18 network, and adds GCN and BR modules. The ECA is added to the spatial path part of BiSeNet. The input image is first processed through spatial and contextual paths. The spatial path includes Conv layer, BN layer, ReLU activation function, and ECA module, which extract spatial features of the image. The context path is composed of an improved ResNet18 network, in which GCN and BR modules are added to optimize the contextual information. The outputs from the two paths are then entered into the attention refinement module, which further optimizes the feature representation. Finally, through the feature fusion module, features from different paths are fused to generate the final road segmentation result. Ultimately, irregular roads are recognized.

### 2.2. Vehicle driving area detection model based on improved bisenet and data dimensionality reduction

**Fig 4:**
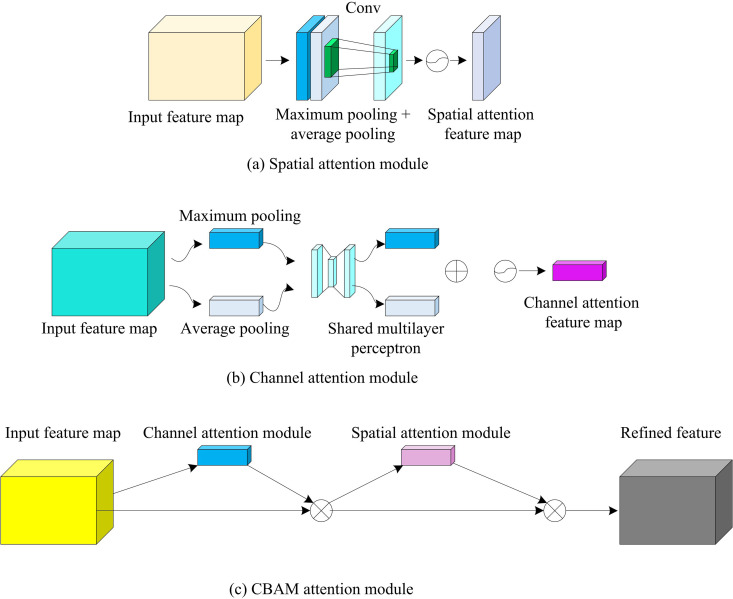
CBAM structure.

Due to the frequent potholes on irregular road surfaces, it is necessary to identify potholes after using the improved BiSeNet based road recognition algorithm to recognize irregular roads. Using effective methods to identify road potholes can help ensure safe driving. Therefore, the YOLOv5 is used to detect potholes. YOLOv5 is an object detection algorithm, which has fast and accurate detection speed and fast inference speed, applied in autonomous driving and object detection [23]. However, the road has many types and varying sizes of potholes, which leads to poor detection performance. Therefore, the research first takes the Convolutional Block Attention Module (CBAM) to perform improvements. CBAM has high computational efficiency, strong generalization ability, and strong interpretability, with extensive applications in image classification and autonomous driving. The CBAM is shown in Fig 4 [24].

From Fig 4, the CBAM has a channel attention mechanism and a spatial attention mechanism. The former combines global AP and max pooling with multi-layer perceptrons and Sigmoid activation functions to assign weights to each channel to highlight important features. The later generates spatial weight maps through channel pooling and convolution operations to emphasize important spatial positions. The channel attention $B_{p}(F)$ is presented by formula (5).


$$\begin{array}{cc} B_{p}(F) & =\sigma (MLP(AvgPool(F))+MLP(MaxPool(F))\\ & =\sigma (O_{1}(O_{0}(F_{avg}^{p}))+O_{1}(O_{0}(F_{max}^{p}))) \end{array}$$ (5)

In equation (5), F and σ are the input feature map and Sigmoid activation function, respectively. $O_{1}$ and $O_{0}$ are weight parameters. $F_{avg}^{p}$ and $F_{max}^{p}$ are AP and Maximum Pooling (MP), respectively. The spatial attention $B_{s}(F)$ is presented in formula (6).


$$\begin{array}{cc} B_{s}(F) & =\sigma (f^{7\times 7}([AvgPool(F);MaxPool(F)]))\\ & =\sigma (f^{7\times 7}[F_{avg}^{s};F_{max}^{s}] \end{array}$$ (6)

In equation (6), $F_{avg}^{s}$ and $F_{max}^{s}$ represent AP and MP. Secondly, the Alpha IoU Loss function replaces the CIoU Loss function to improve the generalization ability. The Alpha IoU Loss function $Los_{\alpha -CloU}$ is represented by formula (7).


$$Los_{\alpha -CloU}=1-IoU^{\alpha }+\frac{\rho^{2\alpha }(l,l^{gt})}{r^{2\alpha }}+(\chi \upsilon {)}^{\alpha }$$ (7)

**Fig 5:**
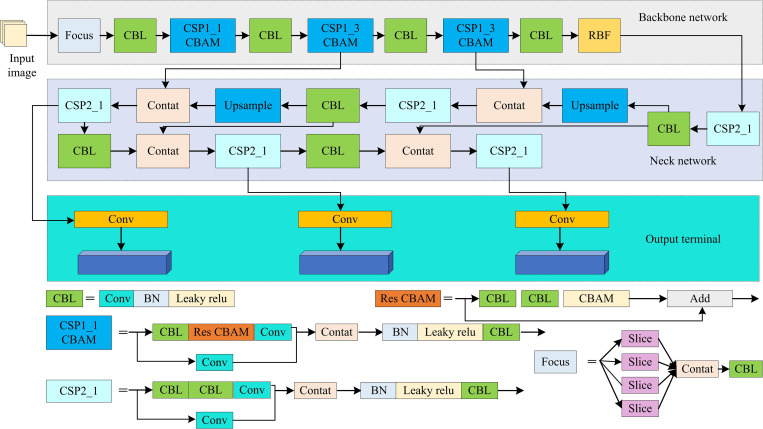
The improved YOLOv5.

In equation (7), α and IoU are the adjustment parameter and the intersection over union ratio between the predicted box and the true box, respectively. ρ(·) and $l^{gt}$ signify the Euclidean distance and the coordinates of the center point of the true box. l is the center point coordinate of the prediction box. r signifies the diagonal length of the smallest closed box. χ is the shape consistency factor. υ signifies the variable used to calculate the shape consistency factor. Then, a Receptive Field Block (RFB) is introduced to optimize the feature extraction of YOLOv5. RFB is inspired by the human visual system, which has advantages such as strong real-time performance and versatility. It is widely used in object detection and road disease detection. The RFB module consists of multiple branch convolutional layers and subsequent expansion pooling or convolutional layers. Each branch contains different convolution kernel sizes and dilation rates to simulate receptive fields of different scales. Finally, the feature maps of all branches are concatenated to form a spatial pooling or convolution array to generate the final feature representation. The RFB, CBAM, and Alpha IoU Loss function are introduced into YOLOv5 to obtain an improved YOLOv5 algorithm. The improved YOLOv5 structure is shown in Fig 5.

**Fig 6:**
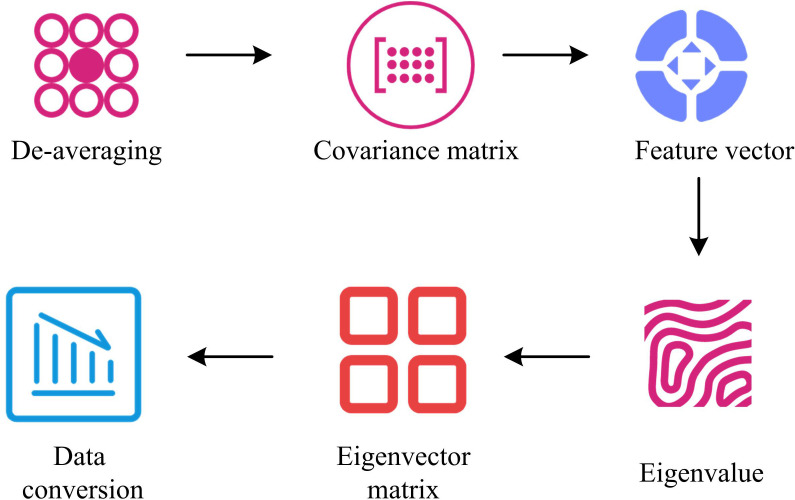
Basic process of the PCA.

From Fig 5, the improved YOLOv5 integrates RFB and CBAM in the backbone network, which enhances the feature extraction capability and better capture detailed information in images. Secondly, the improved YOLOv5 adopts the AlphaIoULoss, which can optimize the prediction accuracy of bounding boxes and reduce the overlap error between predicted boxes and real boxes. The distance between the pothole location and the vehicle is calculated using the grounding point distance measurement method. As this study focuses on identifying irregular road surfaces and detecting potholes while driving, visual sensors are used. Visual sensors operate in real-time during the driving process of a car, capturing image data that contains rich pixel information and has a large data dimension. Therefore, the PCA is introduced for dimensionality reduction. The research chooses to apply the PCA algorithm for image data dimensionality reduction. The key lies in its high computational efficiency when dealing with large-scale datasets, its ability to quickly extract the main features of the data, and its simple implementation [25]. PCA reduces the dimension of the data by identifying the direction with the greatest variance in the data, thereby eliminating redundant information and retaining the most important features [26]. Although PCA is a linear method and may not be able to fully capture all the nonlinear features in the image data, it still has obvious advantages in terms of feature independence and dimensionality reduction efficiency in the preprocessing stage [27]. Although PCA is sensitive to outliers and may lose some information that is important for specific tasks [28]. However, the subject of this study is the road surface detection in the vehicle driving area. During the detection process, the redundant information and image dimensions obtained are relatively high, and the requirements for computational efficiency are extremely high. Common methods such as Autoencoder and t-SNE are not applicable to the current situation. Therefore, it is better to choose PCA for dimensionality reduction. PCA is an unsupervised machine learning technique that has advantages such as reducing data dimensionality, removing noise and redundant information, which is extensively applied in image processing and machine learning. The basic process of PCA is presented in Fig 6 [29].

From Fig 6, the PCA algorithm first performs a de-averaging process, which is to obtain the mean of each feature in the high-dimensional dataset $D\in R^{m\times n}$, which can be represented by formula (8).


$$D=\left[\begin{array}{cccc} {}*20cd_{11} & d_{12} & \cdots & d_{1n}\\ d_{21} & d_{22} & \cdots & d_{2n}\\ \vdots & \vdots & \ddots & \vdots \\ d_{m1} & d_{m2} & \cdots & d_{mn} \end{array}\right]$$ (8)

In equation (8), m represents the number of samples. n is the number of features, with each row and column representing a feature and a sample, respectively. $d_{ij}$ is the i -th observation value of the j -th feature. The average value $\overset{―}{d_{i}}$ of any feature in D can be represented by formula (9).


$$\overset{―}{d_{i}}=1/m\sum_{i}d_{ij}$$ (9)

The value $D^{\prime }$ obtained after de-averaging can be represented by formula (10).


$$D^{\prime }=\left[\begin{array}{cccc} {}*20cd_{11}-\overset{―}{x_{1}} & d_{12}-\overset{―}{x_{2}} & \cdots & d_{1n}-\overset{―}{x_{n}}\\ d_{21}-\overset{―}{x_{1}} & d_{22}-\overset{―}{x_{2}} & \cdots & d_{2n}-\overset{―}{x_{n}}\\ \vdots & \vdots & \ddots & \vdots \\ d_{m1}-\overset{―}{x_{1}} & d_{m1}-\overset{―}{x_{2}} & \cdots & d_{mn}-\overset{―}{x_{n}} \end{array}\right]$$ (10)

Secondly, the covariance matrix T is solved, which can be represented by formula (11).


$$T=\left[\begin{array}{cccc} {}*20c\varepsilon_{11} & \varepsilon_{12} & \cdots & \varepsilon_{1n}\\ \varepsilon_{21} & \varepsilon_{21} & \cdots & \varepsilon_{2n}\\ \vdots & \vdots & \ddots & \vdots \\ \varepsilon_{n1} & \varepsilon_{n2} & \cdots & \varepsilon_{nn} \end{array}\right]$$ (11)

In equation (11), $\varepsilon_{ij}=\varepsilon_{ji}$ represents the covariance between feature i and feature j, where $\varepsilon_{ij}$ can be obtained using equation (12).


$$\varepsilon_{ij}=Cov(D^{\prime }(:,i),D^{\prime }(:,j))=E[(D^{\prime }(:,i)-E(D^{\prime }(:,i)))(D^{\prime }(:,j)-E(D^{\prime }(:,j))]$$ (12)

In equation (12), $D^{\prime }(:,i)$ and $D^{\prime }(:,j)$ signify the i -th and j -th columns, and $D^{\prime }(:,i),D^{\prime }(:,j)\in R^{m\times 1}$. Then, the feature vector and feature value of the covariance matrix are counted. In PCA algorithm, principal components are the parts that contain the majority of feature information, with the largest being the first principal component, followed by the second principal component, etc, to obtain all principal component parts. If $ZC_{1}$ is set as the first principal component, then to obtain $ZC_{1}$, it can be regarded as the value of the projection of all features of $D^{\prime }$ on $ZC_{1}$. This value is the maximum value, as displayed in formula (13).


$$\begin{array}{c} max\frac{\text{1}}{n}\sum_{i=1}^{n}{\left|D^{\prime }(:,i)\cdot ZC_{1}\right|}^{2}\\ {}[10pt]s.t.Z{C_{1}}^{T}ZC_{1}=1 \end{array}$$ (13)

Given the eigenvector $ZC_{1}$, the average value $J(ZC_{1})=\frac{\text{1}}{n}\sum_{i=1}^{n}{\left|D^{\prime }(:,i)\cdot ZC_{1}\right|}^{2}$ is the sum of variances of all features projected onto $ZC_{1}$ in the dataset $D^{\prime }$, where $J(ZC_{1})=\frac{\text{1}}{n}Z{C_{1}}^{T}D^{\prime }{D^{\prime }}^{T}ZC_{1}$. $\frac{\text{1}}{n}Z{C_{1}}^{T}D^{\prime }{D^{\prime }}^{T}$ is the covariance matrix of $D^{\prime }$. Thus, the solution of equation (13) can be transformed, which can be represented by equation (14).


$$\begin{array}{c} maxZC_{1}^{T}TZC_{1}\\ {}[5pt]s.t.ZC_{1}^{T}ZC_{1}=1 \end{array}$$ (14)

A Lagrange function is built to obtain $L_{a}(ZC_{1})=ZC_{1}^{T}TZC_{1}+\psi (1-ZC_{1}^{T}ZC_{1})$, where ψ is the eigenvalue. By taking the derivative of $ZC_{1}$, $TZC_{1}=\psi ZC_{1}$ can be obtained, from which the first principal component is the eigenvector corresponding to the largest eigenvalue among all the largest eigenvalues. If there are several maximum principal components, only the feature vector corresponding to the ξ largest eigenvalues in the covariance matrix needs to be solved to obtain them. Then, the first ξ feature values obtained are sorted in descending order. After combining all the eigenvectors, the eigenvector matrix $Y_{PCA}$ can be obtained, which can be represented by formula (15).


$$Y_{PCA}=[ZC_{1},ZC_{2},\ldots ,ZC_{\xi }{]}^{T}$$ (15)

**Fig 7:**
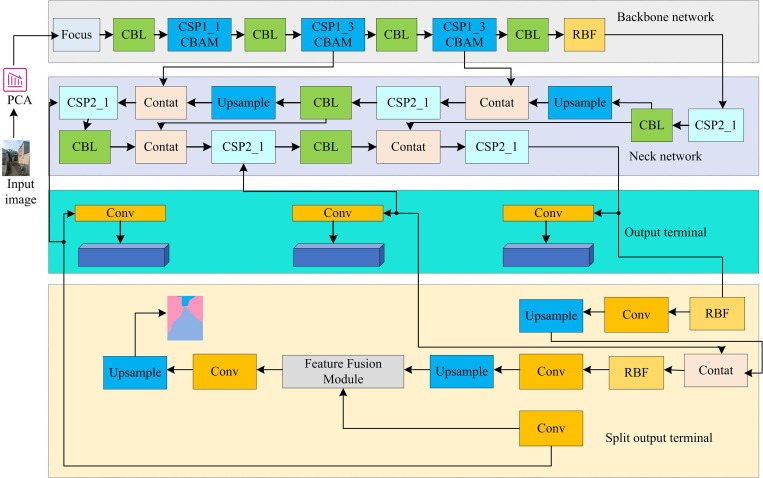
Vehicle driving area detection model based on improved BiSeNet and data dimension reduction (Source from: Photo by author).

Finally, by converting the values obtained from equation (15), the low dimensional representation $V=DY_{PCA}^{T}$ of the reduced data can be obtained. Therefore, the study utilizes a multi-task learning network based on hard parameter sharing to integrate PCA algorithm, improved YOLOv5 algorithm, and vehicle driving road recognition algorithm based on improved BiSeNet. A vehicle driving area detection model on the basis of improved BiSeNet and data dimensionality reduction is built. The model is shown in Fig 7.

**Fig 8:**
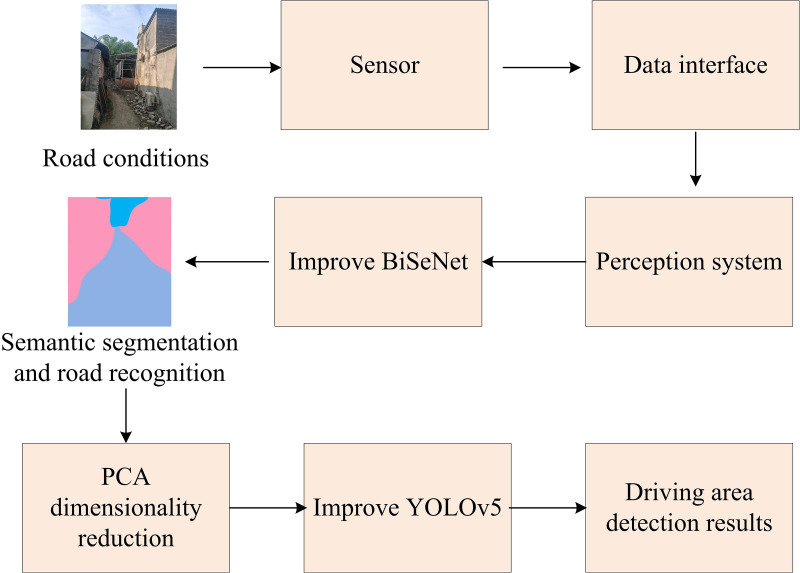
Network structure diagram (Source from: Photo by author).

From Fig 7, the model uses a shared backbone network to obtain the basic features, and then utilizes two branch networks to further characterize these features. The backbone network is an improved YOLOv5 algorithm used to capture key information in images. Two branch networks are used to improve the context path and spatial path of BiSeNet. They are taken to extract spatial and contextual information of input features. Spatial paths are used to extract local features, while contextual paths are used to capture broader environmental information. These two branches process and stack features through up-sampling and down-sampling. After feature extraction, the model uses a feature fusion module to integrate feature maps, and the integrated feature maps are sent to the output. The detection output terminal utilizes the RFB module to enhance the visual receptive field, and then outputs the object detection result through convolution and down-sampling operations. At the detection and segmentation end, the model maintains the encoder and decoder of the original model. In this detection model, the ECA module is used to capture the correlations between channels and enhance the feature expression ability. The size of the input feature map is 224 × 224 × 64, that is, the width is 224 pixels, the height is 224 pixels, and there are 64 channels. The ECA module first compresses the feature map to 1 × 1 × 64 through GAP. The output feature map size of the ECA module is also 1 × 1 × 64. The convolution kernel size used in the ECA module is 1 × 1, and the offset is set to 1 to adjust the ratio between the number of channels and the size of the convolution kernel. The BR module is used to enhance the quality of boundaries and the accuracy of segmentation. Suppose the size of the input feature map is 56 × 56 × 128, that is, the width is 56 pixels, the height is 56 pixels, and there are 128 channels. The BR module extracts the context information through the global convolutional network, and the size of the output feature map is also 56 × 56 × 128. The downsampling and upsampling multiples of the BR module are set to 8, and the feature extraction multiples are 16 times and 32 times respectively. The GCN module is used to combine boundary features with global context information to enhance the accuracy and robustness of boundary detection. The input feature map size is 28 × 28 × 256, that is, the width is 28 pixels, the height is 28 pixels, and there are 256 channels. The output feature map size of the GCN module is also 28 × 28 × 256. The GCN convolution parameters are set to 3 × 1 × 256 × 21 and 1 × 3 × 21 × 21, where 3 represents the width of the convolution kernel, 256 represents the number of channels, and 21 is the depth value of the convolution kernel. When dealing with multimodal data from visual sensors, lidars and millimeter-wave radars, the study first uses the improved BiSeNet network to perform semantic segmentation on image data and extract feature maps of road areas. Meanwhile, voxel mesh filtering sampling is carried out on the point cloud data of the lidar to extract sparse point cloud features. And extract the speed and distance information of the target from the millimeter-wave radar data, and convert it into a format that matches the data of the visual sensor and lidar. Secondly, we concatenate the feature map of the visual sensor, the sparse point cloud feature of the lidar, and the speed and distance feature of the millimeter-wave radar to form a multimodal feature map. On this basis, the attention mechanism is utilized to learn the importance of different modal features, and the weighted processing of the concatenated feature maps is carried out to enhance the expressive ability of the features. Finally, in the decision-making stage of the model, the detection results of different sensors are fused through a weighted average method, and the final detection result is output. Based on the above content, a multi-task learning network based on hard parameter sharing was studied and established to integrate BiSeNet, YOLOv5 and PCA. The structure diagram of this network is shown in Fig 8.

As can be seen from Fig 8, this algorithm acquires road conditions through sensors and transmits them to the vehicle perception system via interfaces. And regional segmentation is carried out by improving the BiSeNet network. After dimensionality reduction using the PCA algorithm, the road surface conditions are identified by improving YOLOv5. This algorithm performs semantic segmentation and road recognition through an improved BiSeNet network. The improved BiSeNet network adds an ECA module in the spatial path part for extracting the spatial features of the image. The context path is composed of an improved ResNet18 network, with GCBN and BR modules added to enhance the context information of the features. The outputs of the two paths are then sent to the attention refinement module, which further optimizes the feature representation. Finally, through the feature fusion module, features from different paths are fused to generate the final road segmentation result.

To further enhance the effect of feature extraction, the RFB and CBAM modules are integrated into the backbone network of YOLOv5 to strengthen the feature extraction capability and enable the network to better capture the detailed information in the image. Furthermore, the improved YOLOv5 adopts the AlphaIoU Loss function, which can optimize the prediction accuracy of bounding boxes and reduce the overlap error between the predicted boxes and the real boxes.

In terms of dimensionality reduction processing, the image data is preprocessed through the PCA algorithm to reduce the dimension of the image data, thereby lowering the computational complexity and improving the operational efficiency of the model. The model shares a backbone network to extract the basic features of the image, and then uses two branch networks to further extract these features respectively. Spatial paths are used to extract local features, while contextual paths are used to capture broader environmental information. These two branches process and stack features through upsampling and downsampling. After feature extraction, the model uses a feature fusion module to fuse feature maps of different scales, and the fused feature maps are sent to the output terminals of detection and segmentation. The detection output terminal uses the RFB module to enhance the field of view receptive field of the feature map, and then through a series of convolution and downsampling operations, finally outputs the target detection result.

## 3. Results

### 3.1. Performance analysis of vehicle driving road recognition algorithm based on improved bisenet

After constructing an improved vehicle driving road recognition algorithm, performance analysis experiments are conducted on it. The experiment uses the PyTorch to design the algorithm. It is updated and optimized using the stochastic gradient algorithm, with an initial learning rate and batch size of 0.001 and 64. The training is conducted 200 times, and over-fitting is avoided through the early stopping method. The data is sourced from the ORFD dataset. This dataset contains a series of types of road damage. The types of damage include long cracks, horizontal cracks, diagonal cracks, depressions, diagonal cracks crocodile cracks and manhole covers. In the data preprocessing stage, enhancement techniques such as random rotation, flipping, scaling and color jitter were adopted to improve the generalization ability of the model. The enhanced data was dimensionally reduced through the PCA algorithm. A total of 28,736 pieces of data were divided into a training set of 70%, a validation set of 20%, and a test set of 10%. To ensure that the model does not lean towards a certain category, have balanced the sample size of each category. All images have been uniformly adjusted to 512 × 512 pixels to ensure the consistency of model input. In addition, the research conducted a detailed statistical analysis of the distribution of various categories in the dataset and ensured that the samples covered multiple scenarios such as rainy days, cloudy days, sunny days, and foggy days, in order to enhance the model’s adaptability to different environmental conditions. To enhance the stability of model evaluation, a K-fold cross-validation strategy was adopted. The dataset was divided into k subsets. Each time, k-1 subsets were used for training, and the remaining subset was used for testing. This process was repeated k times, with a different subset selected as the test set each time, thereby reducing the variance of the results. The experimental comparison algorithms are SegNet, U-Net, and BiSeNet. The experimental comparison indicators include the quantity of frames processed per second, mean Pixel Accuracy (mPA), and Mean IoU (MIoU). The experimental environment is shown in Table 1.

**Table 1:** Specific experimental environment.

Parameter names	Parameter
Processor	Intel Core i9-9880H
Main frequency	4.8GHz
Internal memory	32GB
Hard disk capacity	500GB
Operating system	Ubuntu 18.04
Deep learning framework	Pytorch 1.13.0
GPU	NVIDIA GeForce RTX 3060
Computer language	Python 3.7.16
Data analysis software	Spss23.0

The study first conducts ablation experiments on the improved BiSeNet algorithm, as presented in Table 2.

**Table 2:** Ablation experiment results.

Model	F1 value(%)	AUC	Recall (%)
/	89.27	91.42	94.29
Improvement ResNet18	90.32	96.13	97.23
Improvement ResNet18 + GCN	91.98	96.15	97.28
Improvement ResNet18 + GCN + BRA	94.12	97.28	97.61
Improvement ResNet18 + GCN + BRA + ECA	96.71	97.32	98.08

**Fig 9:**
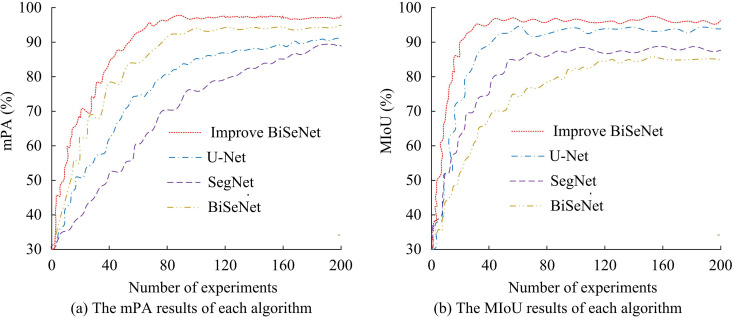
Comparison of mPA and MIoU algorithms.

From Table 2, the F1 value, AUC, and Recall of the algorithm proposed before improvement were 89.27%, 91.42%, and 94.29%, respectively. After sequentially adding ResNet18, GCN, BRA, and ECA, the F1 value, AUC, and Recall of the proposed algorithm reached 96.71%, 97.32%, and 98.08%, respectively. In addition, the information loss rate of the PCA algorithm for dimensionality reduction of 1,478 features was 17.8%. The mAP of the model before dimensionality reduction was 78.5%, and it increased to 81.2% after dimensionality reduction. This indicates that PCA dimensionality reduction not only reduces the computational complexity but also improves the segmentation accuracy of the model. The impact on the accuracy of downstream segmentation tasks, on the one hand, can remove redundant information and retain key information. On the other hand, it helps the model get rid of the interference of unimportant features, thereby enabling it to identify and segment the target more accurately. The above indicates that each module is effective and improves the performance. The MIoU and PA are displayed in Fig 9.

**Fig 10:**
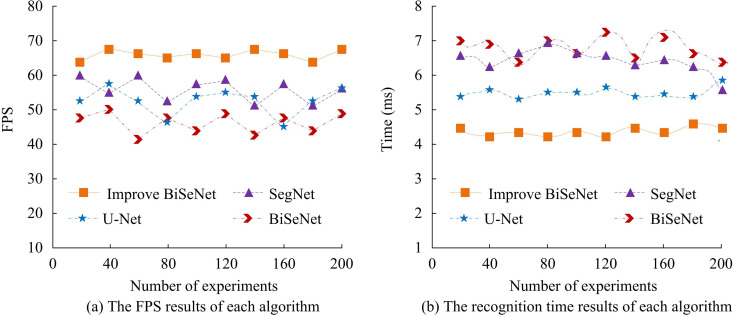
Comparison of frame rate and recognition time for different algorithms.

From Fig 9(a), the improved BiSeNet had an mPA of 97.87%, which was higher than U-Net’s 89.91%, SegNet’s 86.23%, and BiSeNet’s 93.47%. In Fig 9(b), the MIoU of the improved BiSeNet, U-Net, SegNet, and BiSeNet was 97.98%, 91.32%, 83.58%, and 83.21%, respectively. A high mAP indicates high accuracy of the algorithm in object detection tasks, while a high mIoU value indicates good segmentation performance. From the perspectives of mPA and mIoU, the proposed algorithm performs better compared with the comparative algorithms. The number of frames and recognition speed that each algorithm can process images per second are shown in Fig 10.

From Fig 10(a), the improved BiSeNet could process image frames per second at 68.78FPS, U-Net at 60.28FPS, SegNet at 54.67FPS, and BiSeNet at 51.77FPS. The improved BiSeNet had the highest average frame rate per second. According to Fig 10(b), the average recognition time of the improved BiSeNet was 4.45ms, which was below U-Net’s 5.37ms, SegNet’s 6.24ms, and BiSeNet’s 6.93ms. A high number of frames per second processed by the algorithm indicates better real-time performance, while a shorter average recognition time indicates higher efficiency. The improved BiSeNet has better performance compared with the comparative algorithms.

### 3.2. Performance analysis of vehicle driving area detection model

**Fig 11:**
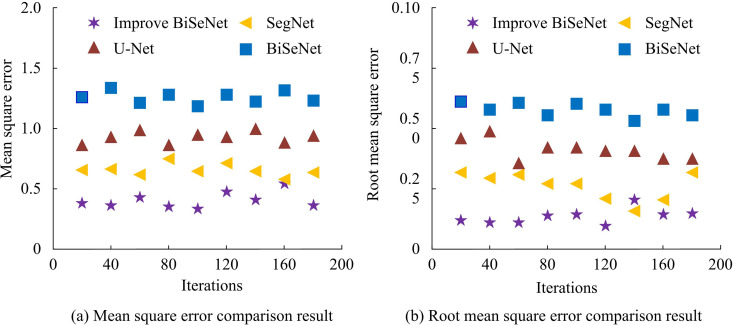
Comparison of Mean Squared Error (MSE) and Root Mean Squared Error (RMSE) for different algorithms.

After analyzing the performance of the vehicle driving road recognition algorithm based on improved BiSeNet, the proposed vehicle driving area detection model is further compared. The comparative models are SegNet, U-Net, and BiSeNet. The model is trained 300 times, with a batch size of 8. Adam is taken as the optimizer, and the initial learning rate and weight decay are both 0.001. The weight allocation for the recognition algorithm and the improved YOLOv5 are 0.95 and 0.05. The experimental environment remains unchanged, and early stopping method is used to avoid over-fitting. The experimental comparison indicators have Mean Square Error (MSE), Root Mean Square Error (RMSE), recall rate, accuracy, loss value, and CPU usage rate. The comparison results of the MSE and RMSE of each model are shown in Fig 11.

**Fig 12:**
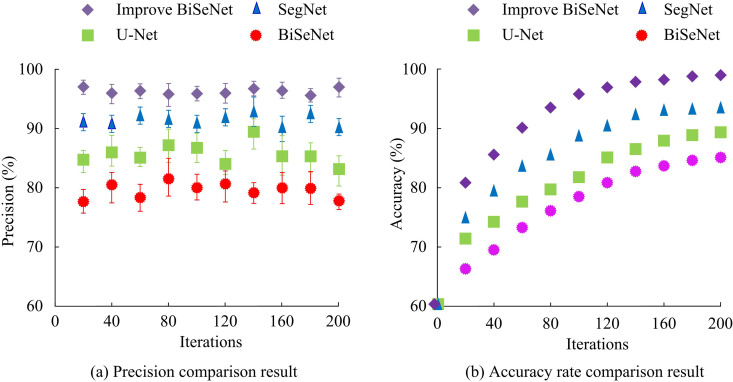
Comparison of precision and accuracy for different algorithmsy.

From Fig 11(a), the average MSE of the improved BiSeNet detection model, SegNet detection model, U-Net detection model, and BiSeNet detection model was 0.476, 0.728, 0.892, and 1.267, respectively, with the proposed model having the lowest average MSE. In Fig 11(b), the average RMSE of the improved BiSeNet detection model proposed in the study was 0.132, which was lower than the 0.174 of the SegNet detection model, 0.368 of the U-Net detection model, and 0.521 of the BiSeNet detection model. A low MSE indicates high accuracy of the model prediction and smaller fluctuations in error. The low RMSE suggests that the mean difference between the predicted and actual is minimal, and the predicted result is closer to the true value. For RMSE and MSE, the detection model performs better compared with the comparative model. The comparison results of precision and accuracy of each model are shown in Fig 12.

In Fig 12(a), the average precision of the improved BiSeNet detection model, SegNet detection model, U-Net detection model, and BiSeNet was 98.97%, 94.26%, 84.27%, and 81.07%, respectively. The proposed detection model had the highest average precision. From Fig 12(b), the accuracy of the improved BiSeNet detection model proposed in the study was 97.66%, significantly higher than the SegNet detection model’s 94.21%, U-Net detection model’s 89.36%, and BiSeNet detection model’s 82.98%. High precision indicates means a stronger ability to tell the difference between positive and negative results. High accuracy indicates stronger detection performance. The proposed detection model outperforms the comparison models on precision and accuracy. The comparison results of the loss values, CPU usage, MIoU, and Pixel Accuracy (PA) are displayed in Table 3.

**Table 3:** Comparison of Loss Values, CPU Usage, Mean Intersection over Union (MIoU), and Pixel Accuracy (PA) for different models.

Model	Loss value	CPU usage	MIoU	PA
Improved BiSeNet	1.38	48.79%	97.62%	97.88%
SegNet	3.26	68.26%	89.97%	90.14%
U-Net	2.97	57.42%	91.58%	92.67%
BiSeNet	4.88	56.54%	90.89%	91.48%

**Fig 13:**
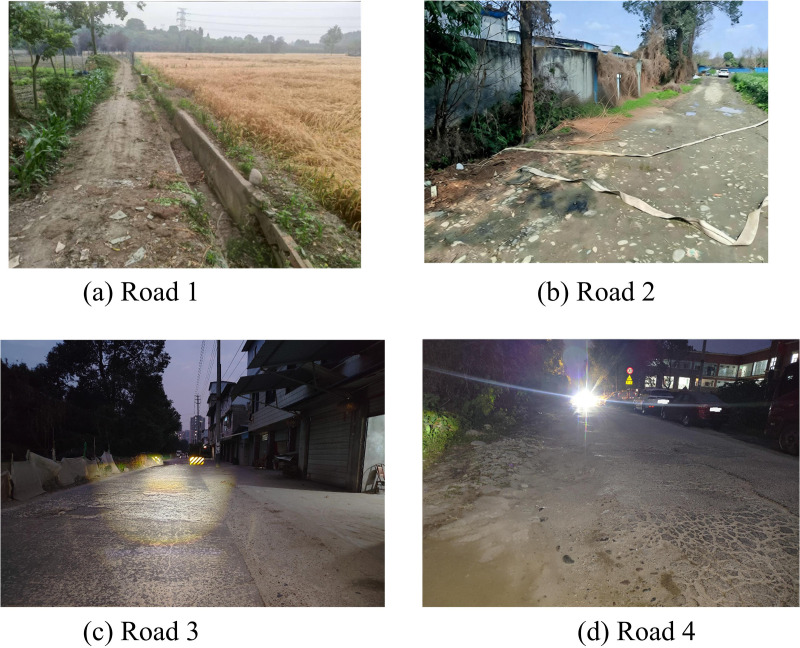
Part of the road pictures in the dataset (Source from: Photo by author).

From Table 3, the proposed improved BiSeNet detection model had a loss value of 1.38, significantly lower than the SegNet detection model’s 3.26, U-Net detection model’s 2.97, and BiSeNet detection model’s 4.88. The CPU usage rates of the improved BiSeNet detection model, SegNet, U-Net, and BiSeNet detection models were 48.79%, 68.26%, 57.42%, and 56.54%, respectively, with the improved BiSeNet detection model having the lowest CPU usage rate. The improved BiSeNet detection model proposed in the study had a MIoU of 97.62% and a PA of 97.88%, significantly higher than SegNet, U-Net, and BiSeNet models. A lower loss value indicates better fitting during the training process. A high MIoU demonstrates that the model has higher segmentation accuracy in image segmentation tasks. A high PA demonstrates that the model performs better in pixel level classification. The proposed detection model performs better on loss value, CPU occupancy, MIoU, and PA compared with comparison model. To verify the application effect of the detection model proposed in the research, 20 irregular roads were randomly selected for shooting, and 2,500 images were captured, with 500 images randomly chosen. And the image is preprocessed. Some of the road images in the dataset are shown in Fig 13.

**Fig 14:**
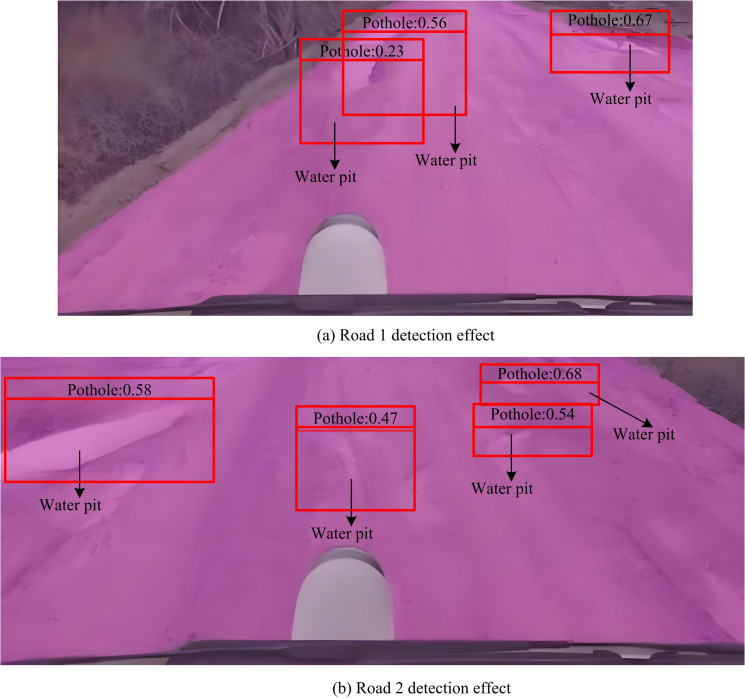
Actual detection effect of model on vehicle driving area.

The preprocessing of the image includes image cropping, scaling and enhancement to meet the input requirements of the model and improve the model’s adaptability to different image conditions. Secondly, a software interface was developed using Python software. Seamlessly integrate the detection model with the vehicle’s perception system through software interfaces. Secondly, developed a software interface using Python software. This interface is responsible for receiving data from vehicle sensors and transmitting it in real time to the detection model for processing. Through this integration, the proposed model can run directly on vehicles, providing real-time road condition detection. The detection effect is shown in Fig 14.

From Fig 14(a), the detection model could not only effectively identify irregular roads during the operation on the road. It could also detect and mark potholes in the driving area. As shown in Fig 14(b), the detection model accurately identified irregular roads while also annotating pothole information during driving on Road 2. The proposed model has practical value. To comprehensively evaluate the performance of the proposed detection model, the study analyzed the detection effects of the proposed detection model and the comparison model under different weather conditions. The comparison indicators include Detection Rate (DR), False Detection Rate (FDR), Average Precision (AP) and Average Recall (AR). The test results under different weather conditions are shown in Table 4.

**Table 4:** Detection results of different models under different weather conditions.

Model	Weather	DR	FDR	AP	AR	References
Improved BiSeNet	Sunny day	97.15%	5.85%	96.47%	91.23%	This study
SegNet		85.67%	10.23%	82.34%	84.56%	[30]
U-Net		88.45%	8.55%	85.12%	87.34%	[31]
BiSeNet		90.32%	7.68%	87.89%	89.12%	[32]
Improved BiSeNet	Rainy day	94.26%	7.26%	89.74%	89.97%	This study
SegNet		90.02%	13.28%	81.21%	82.57%	[30]
U-Net		87.24%	16.85%	80.47%	86.47%	[31]
BiSeNet		88.98%	21.08%	82.58%	85.67%	[32]
Improved BiSeNet	Cloudy day	95.89%.	6.26%	95.41%	90.84%	This study
SegNet		84.55%	7.97%	83.46%	85.62%	[30]
U-Net		83.74%	12.57%	86.71%	84.63%	[31]
BiSeNet		86.38%	11.37%	86.05%	83.27%	[32]
Improved BiSeNet	Foggy day	88.74%	6.82%	91.26%	90.52%	This study
SegNet		81.03%	7.84%	85.28%	84.27%	[30]
U-Net		80.57%	9.34%	86.47%	83.16%	[31]
BiSeNet		81.36%	10.58%	81.09%	84.59	[32]

It can be seen from Table 4 that the values of DR, FDR, AP and AR of the detection model proposed in the study on sunny days are 97.15%, 5.85%, 96.47% and 91.23% respectively. When it rains, the figures are 94.26%, 7.26%, 89.74% and 89.97% respectively. On cloudy days, the figures were 95.89%, 6.26%, 95.41%, and 90.84% respectively. The figures were 88.74%, 6.82%, 91.26% and 90.52% respectively during heavy fog. All are superior to the comparison models. The above results indicate that the detection model proposed in the study is robust under different lighting and weather conditions. To further verify the performance of the proposed detection model, the study analyzed its performance under the input conditions of 1920 × 1080@30fps and when connected to LiDAR. The analysis results are shown in Table 5.

**Table 5:** Performance analysis results.

Model	Frame rate	Power consumption	Satisfies high resolution input	Satisfying LiDAR connectivity	Satisfy the power consumption constraint
Improved BiSeNet	35.7FPS	99.4W	Y	Y	Y
SegNet	15.6FPS	149.8W	Y	Y	Y
U-Net	20.3FPS	183.2W	Y	Y	Y
BiSeNet	30.2FPS	121.4W	Y	Y	Y

As can be seen from Table 5, the Improved BiSeNet detection model proposed in the research performs well under all test conditions, is capable of handling high-resolution input, connecting LiDAR, and has lower power consumption than the constraints. The experimental results show that this model can maintain a processing speed close to real-time without sacrificing too much accuracy. However, as the input resolution and frame rate increase, the computational load will gradually rise, and the requirements for hardware will also increase accordingly. This indicates that although the model performs well under the current conditions, it may require more powerful computing resources or further optimization to meet the power consumption constraints under more demanding conditions.

## 4. Discussion

This research conducted comparative analysis experiments on the road recognition algorithm on the basis of improved BiSeNet, and compared the performance. The road recognition algorithm demonstrated good performance in terms of mPA, MIoU, frame rate per second for image processing, and recognition speed. The mPA of the improved BiSeNet, U-Net, SegNet, and BiSeNet was 97.87%, 89.91%, 86.23%, and 93.47%, with the improved BiSeNet having the highest mPA. The improved ResNet18 network and ECA has enhanced the recognition accuracy. This result is consistent with the improved BiSeNet results designed by L. Teng et al [33]. In the comparative analysis of mIoU and the frame rate performance that can process images per second, the mIoU of the proposed algorithm, U-Net, SegNet, and BiSeNet was 97.98%, 91.32%, 83.58%, and 83.21%, respectively. The frame rates capable of processing images per second were 68.78FPS, 60.28FPS, 54.67FPS, and 51.77FPS, respectively. Among the above results, the improved BiSeNet has the best performance. This indicates that ResNet18, ECA, GCN, and BR have improved the segmentation and recognition performance of the algorithm. This result is matched with the results obtained by T. H. Tsai et al [34]. In addition, the proposed recognition algorithm had a recognition time of 4.45ms, which is superior to the comparison algorithm. This result further proves the superiority of the improved BiSeNet.

Meanwhile, in the comparative analysis of detection models, the mean MSEs of the vehicle driving area detection model, egNet detection model, U-Net detection model, and BiSeNet detection model proposed were 0.476, 0.728, 0.892, and 1.267, respectively. The average RMSEs were 0.132, 0.174, 0.368, and 0.521, respectively. In the above results, the proposed detection model outperforms the comparison model. The improved YOLOv5 has enhanced the detection performance of the model. This result is similar to the findings obtained by J. Zhao et al. in related studies [35]. The average precision of the proposed detection model, SegNet detection model, U-Net detection model, and BiSeNet detection model was 98.97%, 94.26%, 84.27%, and 81.07%, respectively. The accuracy was 97.66%, 94.21%, 89.36%, and 82.98%, respectively. Among the above results, the improved BiSeNet had the best performance. This result indicates that the improved YOLOv5 and PCA algorithms have enhanced the detection accuracy. This is in line with the results obtained by J. Karangwa et al. in 2023 [36]. In addition, in terms of loss value, CPU usage, MIoU, and PA, the detection model proposed in the study was 1.38, 48.79%, 97.62%, and 97.88%, respectively, which was all superior to the comparative model. This result is consistent with the findings obtained by K. Muhammad et al. in 2022 [37]. Finally, the application effect is analyzed. The proposed detection model can accurately identify irregular roads and potholes in roads. The improved BiSeNet has good application value.

## 5. Conclusion

To solve the poor real-time performance and low accuracy of intelligent vehicle driving area detection methods, this study focused on irregular road areas encountered during vehicle driving, and introduced improved ResNet18 network, ECA, GCN, and BR to improve them. A vehicle driving road recognition algorithm based on improved BiSeNet was constructed. The improved YOLOv5 was taken to construct a road pothole detection algorithm. Then, the PCA was taken to make the sensor data less complicated. Finally, combining the three, a vehicle driving area detection method based on BiSeNet and data dimensionality reduction was constructed. The algorithm significantly exceeded the comparison methods on mPA, MIoU, frame rate per second, and recognition speed. Subsequently, performance analysis was conducted on the detection model. The model outperformed the comparison model on mean MSE, mean RMSE, precision, and accuracy. Finally, the application effect of the detection model was analyzed, and the model had good practical value.

The limitation of this study lies in the fact that the model is mainly tested for structured roads, and its performance may be restricted in scenarios without street lamps at night. Future work will explore the adaptability of the model under unstructured roads and complex lighting conditions, as well as enhance the accuracy and robustness of detection through multi-sensor fusion and model lightweighting. In addition, we plan to validate the model in a more diverse range of test environments to enhance its generalization ability and practicality.

## Supporting information

S1 File Minimal data set definition. (DOCX)

## References

[1] Hasanvand M, Nooshyar M, Moharamkhani E, Selyari A. Machine learning methodology for identifying vehicles using image processing. AIA. 2023;1(3):154–62. doi: 10.47852/bonviewaia3202833

[2] Zheng R, Sun S, Liu H, Wu T. Deep Neural networks-enabled vehicle detection using high-resolution automotive radar imaging. IEEE Trans Aerosp Electron Syst. 2023:1–16. doi: 10.1109/taes.2023.3275887

[3] Zhao T, He J, Lv J, Min D, Wei Y. A Comprehensive implementation of road surface classification for vehicle driving assistance: dataset, models, and deployment. IEEE Trans Intell Transport Syst. 2023;24(8):8361–70. doi: 10.1109/tits.2023.3264588

[4] Ai Y, Song R, Huang C, Cui C, Tian B, Chen L. A Real-time road boundary detection approach in surface mine based on meta random forest. IEEE Trans Intell Veh. 2024;9(1):1989–2001. doi: 10.1109/tiv.2023.3296767

[5] Wang Z, Zhan J, Duan C, Guan X, Lu P, Yang K. A Review of vehicle detection techniques for intelligent vehicles. IEEE Trans Neural Netw Learn Syst. 2023;34(8):3811–31. doi: 10.1109/TNNLS.2021.3128968 34986101

[6] Yang S, Chen Y, Shi R, Wang R, Cao Y, Lu J. A Survey of intelligent tires for tire-road interaction recognition toward autonomous vehicles. IEEE Trans Intell Veh. 2022;7(3):520–32. doi: 10.1109/tiv.2022.3163588

[7] Luo T, Chen Y, Luan T, Cai B, Chen L, Wang H. IDS-MODEL: An efficient multitask model of road scene instance and drivable area segmentation for autonomous driving. IEEE Trans Transp Electrific. 2024;10(1):1454–64. doi: 10.1109/tte.2023.3293495

[8] Liu C, Nie T, Du Y, Cao J, Wu D, Li F. A response-type road anomaly detection and evaluation method for steady driving of automated vehicles. IEEE Trans Intell Transport Syst. 2022;23(11):21984–95. doi: 10.1109/tits.2022.3182428

[9] Teng L, Qiao Y, Shafiq M, Srivastava G, Javed AR, Gadekallu TR, et al. FLPK-BiSeNet: federated learning based on priori knowledge and bilateral segmentation network for image edge extraction. IEEE Trans Netw Serv Manage. 2023;20(2):1529–42. doi: 10.1109/tnsm.2023.3273991

[10] Liu Z, Gao Y, Du Q, Chen M, Lv W. YOLO-Extract: Improved YOLOv5 for Aircraft object detection in remote sensing images. IEEE Access. 2023;11:1742–51. doi: 10.1109/access.2023.3233964

[11] Sun Y, Li J, Xu X, Shi Y. Adaptive multi-lane detection based on robust instance segmentation for intelligent vehicles. IEEE Trans Intell Veh. 2023;8(1):888–99. doi: 10.1109/tiv.2022.3158750

[12] Wang Y, Zhang J, Chen Y, Yuan H, Wu C. An automated learning method of semantic segmentation for train autonomous driving environment understanding. IEEE Trans Ind Inf. 2024;20(4):6913–22. doi: 10.1109/tii.2024.3353874

[13] Zhang Z, Xu H, Lin S. Quantizing YOLOv5 for real-time vehicle detection. IEEE Access. 2023;11:145601–11. doi: 10.1109/access.2023.3345220

[14] Yao Z, Liu Q, Xie Q, Li Q. TL-detector: lightweight based real-time traffic light detection model for intelligent vehicles. IEEE Trans Intell Transport Syst. 2023;24(9):9736–50. doi: 10.1109/tits.2023.3267430

[15] Li G, Ji Z, Qu X, Zhou R, Cao D. Cross-domain object detection for autonomous driving: a stepwise domain adaptative YOLO Approach. IEEE Trans Intell Veh. 2022;7(3):603–15. doi: 10.1109/tiv.2022.3165353

[16] Gao G, Xu G, Li J, Yu Y, Lu H, Yang J. FBSNet: A fast bilateral symmetrical network for real-time semantic segmentation. IEEE Trans Multimedia. 2023;25:3273–83. doi: 10.1109/tmm.2022.3157995

[17] Shi X, Yin Z, Han G, Liu W, Qin L, Bi Y, et al. BSSNet: A real-time semantic segmentation network for road scenes inspired from AutoEncoder. IEEE Trans Circuits Syst Video Technol. 2024;34(5):3424–38. doi: 10.1109/tcsvt.2023.3325360

[18] Li X, Li Y, Chen H, Peng Y, Pan P. CCAFusion: Cross-modal coordinate attention network for infrared and visible image fusion. IEEE Trans Circuits Syst Video Technol. 2024;34(2):866–81. doi: 10.1109/tcsvt.2023.3293228

[19] Xue S, Abhayaratne C. Region-of-interest aware 3D resnet for classification of COVID-19 chest computerised tomography scans. IEEE Access. 2023;11:28856–72. doi: 10.1109/access.2023.3260632

[20] Xie Z, Zheng G, Miao L, Huang W. STGL-GCN: Spatial–temporal mixing of global and local self-attention graph convolutional networks for human action recognition. IEEE Access. 2023;11:16526–32. doi: 10.1109/access.2023.3246127

[21] Jia H, Yu S, Yin S, Liu L, Yi C, Xue K, et al. a model combining multi branch spectral-temporal cnn, efficient channel attention, and LightGBM for MI-BCI classification. IEEE Trans Neural Syst Rehabil Eng. 2023;31:1311–20. doi: 10.1109/TNSRE.2023.3243992 37022898

[22] Chen F, Chen L, Kong W, Zhang W, Zheng P, Sun L, et al. Deep semi-supervised ultrasound image segmentation by using a shadow aware network with boundary refinement. IEEE Trans Med Imaging. 2023;42(12):3779–93. doi: 10.1109/TMI.2023.3309249 37695964

[23] Zhang M, Yin L. Solar cell surface defect detection based on improved YOLO v5. IEEE Access. 2022;10:80804–15. doi: 10.1109/access.2022.3195901

[24] Wang W, Tan X, Zhang P, Wang X. A CBAM based multiscale transformer fusion approach for remote sensing image change detection. IEEE J Sel Top Appl Earth Observations Remote Sensing. 2022;15:6817–25. doi: 10.1109/jstars.2022.3198517

[25] Fariz TKN, Basha SS. Enhancing solar radiation predictions through COA optimized neural networks and PCA dimensionality reduction. Energy Reports. 2024;12:341–59. doi: 10.1016/j.egyr.2024.06.025

[26] Sarıkoç M, Celik M. PCA-ICA-LSTM: A hybrid deep learning model based on dimension reduction methods to predict S&P 500 index price. Comput Econ. 2024;65(4):2249–315. doi: 10.1007/s10614-024-10629-x

[27] Salloum S, Alhumaid K, Salloum A, Shaalan K. K-means clustering of tweet emotions: A 2D PCA visualization approach. Procedia Computer Science. 2024;244:30–6. doi: 10.1016/j.procs.2024.10.175

[28] Dorabiala O, Aravkin AY, Kutz JN. Ensemble principal component analysis. IEEE Access. 2024;12:6663–71. doi: 10.1109/access.2024.3350984

[29] Parizad A, Hatziadoniu CJ. Cyber-attack detection using principal component analysis and noisy clustering algorithms: a collaborative machine learning-based framework. IEEE Trans Smart Grid. 2022;13(6):4848–61. doi: 10.1109/tsg.2022.3176311

[30] Hualong Y, Daidou G. Research on double encryption of ghost imaging by SegNet Deep neural network. IEEE Photon Technol Lett. 2024;36(10):669–72. doi: 10.1109/lpt.2024.3379554

[31] Rajamani KT, Rani P, Siebert H, ElagiriRamalingam R, Heinrich MP. Attention-augmented U-Net (AA-U-Net) for semantic segmentation. Signal Image Video Process. 2023;17(4):981–9. doi: 10.1007/s11760-022-02302-3 35910403 PMC9311338

[32] Wang H, Wang B, Zhao T. Shuff-BiseNet: a dual-branch segmentation network for pavement cracks. SIViP. 2024;18(4):3309–20. doi: 10.1007/s11760-023-02993-2

[33] Teng L, Qiao Y. BiSeNet-oriented context attention model for image semantic segmentation. ComSIS. 2022;19(3):1409–26. doi: 10.2298/csis220321040t

[34] Tsai T-H, Tseng Y-W. BiSeNet V3: Bilateral segmentation network with coordinate attention for real-time semantic segmentation. Neurocomputing. 2023;532:33–42. doi: 10.1016/j.neucom.2023.02.025

[35] Zhao J, Wu D, Yu Z, Gao Z. DRMNet: A multi-task detection model based on image processing for autonomous driving scenarios. IEEE Trans Veh Technol. 2023;72(12):15341–55. doi: 10.1109/tvt.2023.3296735

[36] Karangwa J, Liu J, Zeng Z. Vehicle detection for autonomous driving: a review of algorithms and datasets. IEEE Trans Intell Transport Syst. 2023;24(11):11568–94. doi: 10.1109/tits.2023.3292278

[37] Muhammad K, Hussain T, Ullah H, Ser JD, Rezaei M, Kumar N, et al. Vision-based semantic segmentation in scene understanding for autonomous driving: recent achievements, challenges, and outlooks. IEEE Trans Intell Transport Syst. 2022;23(12):22694–715. doi: 10.1109/tits.2022.3207665

