# Supplementary material for: Vehicle driving area detection and sensor data preprocessing based on deep learning
Source: PLoS One. 2025 Dec 16;20(12):e0337722. doi: 10.1371/journal.pone.0337722 (PMC12707679; doi:10.1371/journal.pone.0337722)
Supplement: S1 File — (DOCX) [file pone.0337722.s001.docx]

The data in Figure 9

| Algorithm | Number of mPA experiments | | | | | The number of MIoU experiments | | | | |
| --- | --- | --- | --- | --- | --- | --- | --- | --- | --- | --- |
|  | 40 | 80 | 120 | 160 | 200 | 40 | 80 | 120 | 160 | 200 |
| Improved BiSeNet | 81.82% | 97.89% | 97.86% | 97.87% | 97.87% | 97.13% | 97.12% | 97.87% | 97.88% | 97.98% |
| U-Net | 67.34% | 80.36% | 86.78% | 88.76% | 89.91% | 89.88% | 90.24% | 90.88% | 91.31% | 91.32% |
| SegNet | 51.96% | 70.02% | 77.64% | 82.68% | 86.23% | 80.16% | 82.16% | 83.56% | 83.59% | 83.58% |
| BiSeNet | 78.87% | 90.03% | 91.34% | 93.46% | 93.47% | 74.59% | 75.68% | 82.19% | 83.25% | 83.21% |

The data in Figure 10

| Algorithm | Number of FPS experiments | | | | | The number of Time experiments | | | | |
| --- | --- | --- | --- | --- | --- | --- | --- | --- | --- | --- |
|  | 40 | 80 | 120 | 160 | 200 | 40 | 80 | 120 | 160 | 200 |
| Improved BiSeNet | 67.23PFS | 66.87FPS | 67.68FPS | 68.74FPS | 68.78FPS | 4.48ms | 4.47ms | 4.28ms | 4.52ms | 4.45ms |
| U-Net | 58.13FPS | 49.98FPS | 59.99FPS | 49.98FPS | 60.28FPS | 5.26ms | 5.56ms | 5.47ms | 5.36ms | 5.37ms |
| SegNet | 53.64FPS | 51.16FPS | 58.59FPS | 56.13FPS | 54.67FPS | 6.32ms | 6.93ms | 6.47ms | 6.38ms | 6.24ms |
| BiSeNet | 50.01FPS | 49.91PFS | 50.02FPS | 50.01FPS | 51.77FPS | 6.97ms | 6.94ms | 7.34ms | 7.28ms | 6.93ms |

The data in Figure 11

| Algorithm | Number of MSE experiments | | | | | The number of RMSE experiments | | | | |
| --- | --- | --- | --- | --- | --- | --- | --- | --- | --- | --- |
|  | 40 | 80 | 120 | 160 | 200 | 40 | 80 | 120 | 160 | 200 |
| Improved BiSeNet | 0.471 | 0.492 | 0.512 | 0.524 | 0.476 | 0.124 | 0.142 | 0.103 | 0.136 | 0.132 |
| U-Net | 0.913 | 0.892 | 0.908 | 0.884 | 0.892 | 0.501 | 0.482 | 0.361 | 0.356 | 0.368 |
| SegNet | 0.529 | 0.702 | 0.698 | 0.886 | 0.728 | 0.263 | 0.246 | 0.167 | 0.178 | 0.174 |
| BiSeNet | 1.472 | 1.383 | 1.261 | 1.117 | 1.267 | 0.519 | 0.517 | 0.528 | 0.589 | 0.521 |

The data in Figure 12

| Algorithm | Number of precision experiments | | | | | The number of accuracy experiments | | | | |
| --- | --- | --- | --- | --- | --- | --- | --- | --- | --- | --- |
|  | 40 | 80 | 120 | 160 | 200 | 40 | 80 | 120 | 160 | 200 |
| Improved BiSeNet | 98.94% | 98.93% | 98.99% | 98.98% | 98.97% | 87.78% | 92.65% | 96.78% | 97.58% | 97.66% |
| U-Net | 85.14% | 85.23 | 82,21% | 83.37% | 84.27% | 72.79% | 79.98% | 82.36% | 87.79% | 89.36% |
| SegNet | 91.16% | 94.02% | 94.31% | 9189% | 94.26% | 89.36% | 83.39% | 90.02% | 92.68% | 94.21% |
| BiSeNet | 80.32% | 81.16% | 81.87% | 79.97% | 81.07% | 68.93% | 76.58% | 80.12% | 81.17% | 82.98% |
